# Another Look at the Contribution of Oral Microbiota to the Pathogenesis of Rheumatoid Arthritis: A Narrative Review

**DOI:** 10.3390/microorganisms10010059

**Published:** 2021-12-28

**Authors:** Jean-Marie Berthelot, Octave Nadile Bandiaky, Benoit Le Goff, Gilles Amador, Anne-Gaelle Chaux, Assem Soueidan, Frederic Denis

**Affiliations:** 1 Rheumatology Unit, Nantes University Hospital, Place Alexis Ricordeau, CEDEX 01, 44093 Nantes, France; jeanmarie.berthelot@chu-nantes.fr (J.-M.B.); benoit.legoff@chu-nantes.fr (B.L.G.); 2 Division of Fixed Prosthodontics, University of Nantes, 1 Place Alexis Ricordeau, 44042 Nantes, France; octave.bandiaky@chu-nantes.fr; 3 Department of Dental Public Health, Faculty of Dental Surgery, University of Nantes, 44093 Nantes, France; gilles.amador@univ-nantes.fr; 4 Nantes Teaching Hospital, 44000 Nantes, France; anne-gaelle.chaux@univ-nantes.fr; 5 Department of Oral Surgery, Faculty of Dental Surgery, University of Nantes, 44000 Nantes, France; 6 Department of Periodontology, Faculty of Dental Surgery, UIC 11, Rmes U1229, CHU de Nantes, 44000 Nantes, France; assem.soueidan@univ-nantes.fr; 7 Tours Teaching Hospital, 37000 Tours, France

**Keywords:** rheumatoid arthritis, oral bacteria, *Porphyromonas gingivalis*, dysbiosis, HLA, synovium, translocation, gut permeability, NETosis, periodontitis

## Abstract

Although autoimmunity contributes to rheumatoid arthritis (RA), several lines of evidence challenge the dogma that it is mainly an autoimmune disorder. As RA-associated human leukocyte antigens shape microbiomes and increase the risk of dysbiosis in mucosae, RA might rather be induced by epigenetic changes in long-lived synovial presenting cells, stressed by excessive translocations into joints of bacteria from the poorly cultivable gut, lung, or oral microbiota (in the same way as more pathogenic bacteria can lead to “reactive arthritis”). This narrative review (i) lists evidence supporting this scenario, including the identification of DNA from oral and gut microbiota in the RA synovium (but in also healthy synovia), and the possibility of translocation through blood, from mucosae to joints, of microbiota, either directly from the oral cavity or from the gut, following an increase of gut permeability worsened by migration within the gut of oral bacteria such as *Porphyromonas gingivalis*; (ii) suggests other methodologies for future works other than cross-sectional studies of periodontal microbiota in cohorts of patients with RA versus controls, namely, longitudinal studies of oral, gut, blood, and synovial microbiota combined with transcriptomic analyses of immune cells in individual patients at risk of RA, and in overt RA, before, during, and following flares of RA.

## 1. Introduction

The dysbiotic oral microbiota observed in rheumatoid arthritis (RA) is not necessarily causal [1]. The dysbioses reversed by treatments may just be the consequences of inflammation [2,3]. Even some oral dysbioses persisting after treatment may not directly contribute to RA pathogenesis, but just be linked to systemic factors shared with RA, such as genetics, which shape the periodontal microbiota of RA (up to dysbiosis) independently of their contribution to RA pathogenesis [4]. The importance of the genetic background in periodontitis and arthritis is illustrated by the observation that in other settings, such as the collagen-induced arthritis mouse model, oral inoculation with *Porphyromonas gingivalis* (*Pg*) and *Aggregatibacter actinomycetemcomitans* (*Aa*) (i.e., the two bacteria suspected so far to contribute most to RA pathogenesis) did not exacerbate alveolar bone loss, and had no effect on the severity of arthritis [5].

Nevertheless, a larger body of evidence supports the contribution of oral bacteria such as *Pg* to the pathogenesis of at least a subset of human RA, although it may depend on the genetic background of the host. That evidence is synthetized in Table 1.

**Figure 1:**
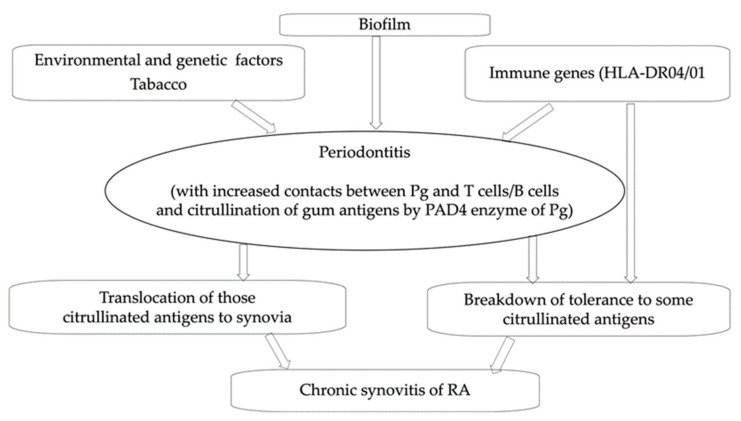
Former hypothesis of contribution of oral bacteria to RA pathogenesis.

How those oral bacteria trigger RA is still uncertain. So far, the most popular hypothesis has been (i) that RA is purely an autoimmune disease, mostly driven by a breakdown of tolerance to citrullinated peptides, promoted by certain sequences of the third hypervariable region of human leukocyte antigen (HLA) class II major histocompatibility molecules D04/01 (the so called “shared epitope” sequences) [1]; (ii) that *Pg* could trigger citrullination of peptides in gingival tissues and stimulate T and B autoreactive clones, which could subsequently recognize similar peptides/antigens in joints [6]. Indeed, *Pg* produces an enzyme, peptidylarginine deiminase (PAD), secreted not only by outer membrane vesicles but also in a soluble form; (iii) that systemic injury by free toxins of oral pathogens, and systemic inflammation caused by those soluble antigens, may further modulate RA activity [7] (Figure 1). Indeed, although periodontal therapy does not have a significant effect on systemic inflammation [8], nonsurgical periodontal treatment seems partly effective in improving periodontal disease, reducing the presence of periodontal pathogens, and improving the RA clinical status (more in patients with RA periodontitis than in patients with RA without periodontitis [9]).

**Table 1:** Clues suggesting a contribution of oral–gingival microbiota to the contribution of rheumatoid arthritis.

Clinical and In Vitro Studies					
Author, Year	Objective	Patients (Mean Age)	Study Design	Study Group	Main Results
Rotsein, I. and Katz, J., 2021 [10]	To assess the prevalence of periapical abscesses in patients with RA, and to evaluate the effects of commonly used antirheumatic medications on such prevalence	NR	A cross-sectional study	Patients with RA were included and data of diagnosis of RA antirheumatic medications, and the presence of periapical abscess were recorded.	Statistically higher prevalence of periapical abscesses in patients with RA than in the general patient population (OR: 2.60) (*p* < 0.0001). Treatment with TNF-α inhibitors may lower the prevalence of periapical abscesses in such patients
Xiao, F. et al., 2021 [11]	To explore the correlation between RA and PD	631 (40.93)	A cross-sectional comparative study	307 patients with RA (RA group) and 324 healthy individuals (control group) were selected, the incidence of PD in both groups was analyzed, and ELISA was used to detect the IL-1β and TNF-α levels in the (GCF) of both groups	The prevalence of PD in the RA group was significantly higher than that in the control group and increased with age and disease duration in patients with RA. The presence of RA can increase risk of PD occurrence and is positively correlated with levels of IL-1β and TNF-α in the GCF.
González, D.A. et al., 2021 [12]	To identify clinical and/or serological variables in patients with RA that are associated with their periodontal severity	110 (48.5)	A cross-sectional study	Subjects with RA and chronic periodontitis were included. RA clinical parameters and rheumatoid factor, presence of bone erosions, and rheumatic nodules) and corticosteroid therapy were considered. Periodontitis was evaluated according to the American Academy of Periodontology (1999).	Periodontitis severity is associated with higher RA disease activity (DAS-284.1; OR: 51.4; 95%, CI 9.4–281.5), longer disease duration, and rheumatoid nodules (OR: 6.; 95% CI: 1.3–31.6).
Moentadj, R. et al., 2021 [13]	To study the oral microbiota in a prospective cohort of patients with RA, FDR, and HC, then to genomically and functionally characterize streptococcal species from each group to understand their potential contribution to RA development.	222 (45.7–53.5)	A cross-sectional comparative study	116 patients with RA, 63 FDR, and 43 HC were included; salivary samples were taken by tongue swabbing for analysis of microbial dysbiosis by using 16S rRNA gene sequencing technology	Dysbiosis-associated periodontal inflammation and barrier dysfunction may permit arthritogenic insoluble pro-inflammatory pathogen-associated molecules, such as streptococcal cell walls, to reach synovial tissue. Streptococcus species can also be enriched in the oral cavity of some RA and their FDRs, and are the source of peptidoglycan-polysaccharide polymers that can induce arthritis in mice
Lehenaff, R. et al., 2021 [14]	To analyze the subgingival microbiome of both shallow (health-associated) and deep (disease-associated) subgingival sites in patients with RA, and then compare them with non-RA household controls.	18 (53.2–55.1)	A case-control study	RA (n = 8) ok and non-RA (n = 10) subjects were recruited and subgingival plaque samples from both shallow (periodontal health-associated, probing depth ≤3 mm) and deep subgingival sites (periodontal disease-associated, probing depth ≥4 mm) were collected. RA subjects also had rheumatological evaluation. Plaque community profiles were analyzed using 16 S rRNA sequencing.	Lack of consistency of gingival microbiomes in RA may relate to numerous explanations: differences in RA subtypes, timing of sampling (RA onset or flares, versus RA in remission), treatments given, and how subgingival plaques are sampled: samples were collected from both shallow (periodontal health-associated, probing depth ≤3 mm) and deep subgingival sites (periodontal disease-associated, probing depth ≥4 mm). In RA, the microbiomes of deep and shallow sites in patients with RA were more similar to each other.
Arévalo-Caro, C. et al., 2021 [15]	To determine the relationship between titres of anti-*Porphyromonas gingivalis* (*P. gingivalis*) antibody andthe RA, HLA-DRB1 susceptibility region associated with SE using the Gregersen’s and de Vries’s classification methods.	100	A case-control study	Results of IgG1 and IgG2 anti-*P. gingivalis* antibodies, ACPA, diagnosis for RA, and PD, and a genetic study of the HLA-DRB1 region were obtained from 50 patients with RA and 50 control individuals.	Although no association was found between SE and anti-*P. gingivalis* antibodies; according to the de Vries’s classification, an association existed between HLA-DRB1 neutral alleles, and high titres of IgG anti-*P.gingivalis* antibodies for RA, focusing on novel associations between *P.gingivalis* and RA. NETosis or intracellular infections by gut pathobionts may also contribute to citrullination. This could explain why anti-Pg antibodies were not clearly associated with SE or ACPAs in patients with RA.
Manoil, D. et al., 2021 [16]	To examine potential correlations between detached subgingival bacteria collected in GCF and RA parameters.	149 (34.95–48.04)	A case-control study	Patients with RA (n = 52) and patients with BD, (n = 40) as another systemic inflammatory disease, were studied along with a systemically healthy control group (HC; n = 57). Full mouth periodontal parameters were recorded. RA activity was assessed using the 28-joint DAS-28. RFs-IgM and -IgA were measured using ELISA. GCF samples were investigated by using fluorescent in situ hybridization for 10 different bacterial taxa.	Altogether, although RA and ACPAs could also be driven by other triggers such as gut bacteria, the PPAD of *Pg* might contribute to ACPAs in a subset of patients with RA. *Pg* displayed significant correlations with not only plaque scores and bleeding on probing, but also with RF-IgA. Patients with RA displaying RF-IgA levels >75 IU/mL exhibited fivefold more abundant *Pg* levels than those with levels below this threshold did. This association with RF-IgA levels appeared even more pronounced, by sixfold more *Pg* levels (*p* = 0.025), in patients with a DAS-28 score >3.2, indicative of being moderately/very active.
Peng, H.Y. et al., 2020 [17]	To determine the role of *Pg* in RA and to identify novel therapeutic targets for auto-inflammatory diseases.	238	A case-control study	Serum samples were obtained from patients with RA (n = 155), PD subjects (n = 48), and HCs (n = 35). The profile of antibody response to gingipain RgpA-specific domains, a cysteine protease produced by *Pg*, was determined in all included patients’ sera, and the potential protective effects of RgpA domains in an experimental arthritis model were also tested.	The pathobiont *Pg* is the bacteria detected most often in patients with both PD and RA, especially in smokers; smoking also strongly worsens PD and RA. Several lines of evidence suggest that *Pg* might contribute to some PD and RA pathogenesis: *Pg* produces cysteine proteases, such as gingipain RgpA, endowed with the potential to induce significant bone loss in animal model systems and in patients in either alveolar or subchondral bone.
Frid, P. et al. 2020 [18]	To characterize the salivary oral microbiome associated with JIA, and correlate it with the disease activity, including gingival inflammation	93 (12.6)	A case-control study	Patients with JIA (n = 59) and healthy controls (HC; n = 34) were recruited, and microbiome profiling of saliva samples was performed by sequencing the V1 V3 region of the 16S rRNA gene.	No differences were found in alpha and beta diversity of oral bacteria among children with JIA and HC. Several taxa associated with chronic inflammation were found to be associated with JIA and disease activity.
Lopez-Oliva, I. et al., 2018 [19]	To characterize the periodontal microbiome in periodontally healthy individuals with and without RA, using next-generation sequencing	41 (36–60)	A case-control study	Patients with RA (n = 22) and non-RA controls (n = 19) were recruted. All participants were periodontally healthy. Subgingival plaque samples were collected and analyzed using 16S rDNA sequencing.	In periodontally healthy individuals with RA, the oral microbiome is enriched for pro inflammatory organisms, and those capable of producing substantial amounts of citrulline (pro-antigenic).
Dong, X.H. et al., 2018 [20]	To present evidence showing that *P. gingivalis* OMVs promote the mucosal transmission of HIV-1	NA	In vitro	The host cells were co-incubated with HIVNL4.3 alone, HIV/*P. gingivalis* 33,277 vesicle complexes, or HIV/KDP 128 vesicle complexes for 30 min. Cell-free complexes were removed by washing the cells with PBS, and the cells were then fixed, permeabilized, immunostained, and analyzed using confocal microscopy.	RANK overexpression and an increase in the ratio of RANK-L to osteoprotegerin was observed in both PD and RA, with a high level of RANK-L expression on gingival B cells, most notably those capable of recognizing *Pg*. Infection with the virus worsens both periodontitis and RA, which act by promoting the growth of organisms such as *Pg*. Reciprocally, *Pg* could foster infection of oral keratinocytes by viruses such as EBV and CMV, as *Pg* OMVs promote the mucosal transmission of viruses
Stobernack, T. et al., 2018 [21]	To identify possible functions of PPAD in the periodontal environment.	NA	In vitro	Human paraffin-embedded gingival tissues were collected from patients with *Pg*-colonized periodontitis. Deparaffinization of 5 m sections was performed by several xylene, ethanol, and water washes. Endogenous peroxidase activity was inhibited by the addition of hydrogen peroxide in methanol, followed by blocking of nonspecific antibody binding with 1% bovine serum albumin and 1% normal goat serum in PBS.	PPAD induces the citrullination of various autoantigens. The rationale for this *Pg*-induced citrullination may be linked to the ability of PPAD to citrullinate the histone H3, thereby facilitating bacterial escape from NETs, and immune evasion of *Pg*. The PAD enzyme, detectable in the gingiva of patients with PD, neutralizes human innate immune defenses at three other distinct levels: bacterial phagocytosis, capture in NETs, and killing by the lysozyme-derived cationic antimicrobial peptide LP9
Zhang, X. et al., 2015 [2]	To characterize the oral and gut microbiomes in patients with RA compared to heathly controls.	191 (18–65)	A case-control study	A metagenomic shotgun sequencing and a metagenome-wide association study of fecal, dental, and salivary samples from treatment-naive individuals with RA (n = 94), and HC (n = 97) was conducted.	Compared to heathly controls, dysbiosis was detected in the gut and oral microbiomes of patients with RA, but it was partially resolved after RA treatment. Alterations in the gut, dental, or saliva microbiome distinguished individuals with RA from HC.
Scher, J.U. et al., 2013 [22]	To determine if particular intestinal bacteria are associated with RA	114 (42.4–50)	A cross-sectional comparative study	Subjects with NORA (n = 44), chronic, treated RA (n = 26), PsA (n = 16), and HCs (n = 28) were included, and 16S sequencing (of the 16S gene (regions V1–V2, 454 platform) on 114 stool samples from patients with RA and controls, and shotgun sequencing on a subset of 44 such samples were performed.	Many cases of RA may later be induced by pathobionts from gut microbiota, such as Prevotella *Copri*, indicating a potential role of this bacterium in the pathogenesis of RA.
**Review articles**					
Alghamdi, M.A., 2021 [23]	To highlight the effect of both gut and oral microbiota dysbiosis on the development of RA, as well as to discuss how the alteration in microbiota composition leads to the overgrowth of some bacterial species entangled in RA pathogenicity.	NA	Review	NA	*Pg*-induced citrullination may contribute to the occurrence of antibodies to citrullinated peptides (ACPA), the most specific signature of RA, especially in patients with the SE HLA-DRB1-04/01
Nik-Azis, N.M. et al., 2021 [24]	To discuss how RA and specifically ACPA-positive RA link to PD, and to appraise the epidemiologicalevidenceonthe relationship between ACPA-positive RA and PD	NA	Review	Articles were searched following the PRISMA guidelines across the Medline, Web of Science, Scopus, and Cochrane Library databases	The severity manifestations of periodontal disease did not differ much in patients with RA with and without ACPA
Chu, X.J. et al., 2021 [25]	To describe a possible relationship between RA and the microbiome of the oral cavity and gut.	NA	A systematic review	A bibliographic search was performed in three databases (EMBASE, Cochrane Library, and PubMed) from inception to 7 June 2020 to identify case control studies that compared the oral and gut microbiomes in adult patients with RA to those of controls	Of 26 articles reviewed, ≥3 articles reported decreased Streptococcus and Haemophilus contrasting with increased Prevotella in the oral cavity of patients with RA compared with heathy controls. In addition, some Prevotella species, including *P. histicola* and *P. oulorum*, showed increased trends in oral cavites of patients with RA, compared with HCs.
Gonzalez-Febles, J. et al., 2021 [26]	To update existing information on the epidemiological association between RA and PD and the biological mechanisms linking these two diseases. To determine whether treatment of PD could influence the initiation and progression of RA.	NA	Review	PubMed, Medline, and Cochrane Library	There was a clear association between PD and RA, with ORs ranging from 1.82 to 20.57 and patients with RA having a high prevalence of PD and tooth loss. The presence of both periodontal inflammation and high numbers of periodontopathic bacteria (*Pg* and *Aa*) have been associated with the onset of RA and increased RA disease activity. Nonsurgical periodontal therapy seems to play a role in the control of RA disease activity.
Zorba, M. et al. 2020 [27]	To review current literature on the possible role of the oral microbiome in the pathogenesis of autoimmune diseases.	NA	Review	PubMed, Medline, Research Gate, and Google Scholar	Oral dysbiosis has also been reported in other adult disorders sometimes overlapping with RA (Sjögren’s syndrome, systemic lupus erythematosus, RA, BD, Crohn’s disease, and psoriasis).
Berthelot, J.M and Le Goff, B., 2010 [28]	To assess the prevalence of PD in patients with RA.	NA	Review	PubMed, Medline, and EMBASE	Modest but increased prevalence of PD among patients with RA compared to the general population, unrelated to secondary Sjögren’s syndrome. Indeed, the prevalence of the SE HLA-DRB1-04 is increased in both RA and PD, and exacerbated T-cell responsiveness with high tissue levels of IL-17 and exaggerated B-cell/plasma cells responses are found in both the synovium and gingival tissue affected with PD.

Abbreviations: ACPA: anticitrullinated peptides antibodies; BD: Behcet’s disease; CMV: cytomegalovirus; CI: confidence interval; DAS; Disease Activity Score; EBV: Epstein–Barr virus; FDR: first-degree relative; GCF: gingival crevicular fluid; HC: healthy controls; IgG1: immunoglobulin G1; IgG2: immunoglobulin G2; IL: interleukin; JIA: juvenile idiopathic arthritis; NORA: new-onset rheumatoid arthritis; OMVs: outer-membrane vesicles; OR: odds ratio; PBS: phosphate-buffered saline; PD: periodontal disease; *Pg*: *Porphyromonas gingivalis*; PPAD: porphyromonas peptidylarginine deiminase; PsA: psoriatic arthritis; RA: rheumatoid arthritis; RANK: receptor activator of nuclear factor κ B; RANK-L: receptor activator of nuclear factor κ B ligand; RFs: rheumatoid factors; rRNA: ribosomal ribonucleic acid; SE: shared epitope; TNF: tumor necrosis factor. However, another look at the contribution of oral bacteria to RA is welcome, as the hypothesis that some oral bacteria such as *Pg* could elicit autoimmunity just because of cross-reactivity between citrullinated peptides in the synovium and other peptides hypercitrullinated in gingiva by the PAD of *Pg* does not fit well with numerous observations made by rheumatologists.

Although autoimmunity obviously contributes to the pathophysiology of most cases of RA, several lines of evidence challenge the dogma that RA is purely an autoimmune disorder driven only by autoreactive T and/or B cells, especially at its onset: (1) arthritis is not a cardinal feature of purely human autoimmune diseases (IPEX and APECED syndromes) [29,30]; (2) RA frequently overlaps with conditions such as psoriatic arthritis and spondyloarthritis (SpA), which are nowadays considered as more driven by innate cells than by T or B lymphocytes [31]; (3) although some peptides found in RA synovium are targeted by T cells and cross-react with various bacteria of the gut microbiota, those T cells might remain driven by those microorganisms, and are reactivated during flares; (4) the association of RA and SpA with HLA could result more from the influence of HLA on RA microbiomes [32] than from the poor elimination of some autoreactive clones specific for citrullinated peptides in patients with at-risk HLA; moreover, shared epitope sequences of HLA-DR04/01 are not linked to immune responses against specific citrullinated peptides [1]; (5) anticitrullinated peptide antibodies (ACPAs), the most specific marker of RA, first elicited in mucosae, are present years before any synovitis, but are also lacking in 25% of RA cases [33]. ACPAs are frequently found in relatives without arthritis [34], and have, like rheumatoid factor (RF), a low predictive value for RA in the general population [35]; (6) titers of ACPAs poorly correlate with RA activity at the individual level; (7) the uneven patterns of synovitis in many cases of RA (some joints being severely affected in a single patient, whereas their neighbors are perfectly spared) would better fit with the initial metastasis of various microbial antigens from mucosae to joints as triggers for those conditions, especially as Whipple’s disease induced by creeping infection of synovia by the gut bacterium *Tropheryma whipplei* often mimics RA [36]; (8) palindromic rheumatisms can antedate both Whipple’s disease and RA by several years, but only a minority of palindromic rheumatisms develop RA over time in population-based studies [37]; (9) fluctuations of RA disease activity are frequent, up to frank flares [38], which do not fit with pure autoimmune processes, and suggest the contribution of external triggers such as microorganisms. Similarly, spontaneous improvements and remissions do not match the lasting breakdown of T or B cell tolerance to some citrullinated antigens in joints as the main explanation for the RA process; (10) drugs targeting presenting cells are more effective than drugs targeting T cells.

Altogether, although T and B autoimmune responses certainly worsen RA in most patients, RA might, like other inflammatory rheumatisms, be initiated by lasting epigenetic changes of long-lived presenting cells/stem cells within some synovia [39], induced by repeated exposition to various microbial dangers [40]. In other words, RA might be a kind of reactive arthritis induced by the repeated translocation from mucosae to some joints of mucosal bacteria/fungi (either still alive or already dead). The differences between RA and disorders previously called “reactive arthritis” may be that (i) the microorganisms triggering RA could belong to the poorly cultivable gut, lung, and/or oral microbiota, rather than to the more pathogenic set of numerous pathogenic mucosal bacteria and yeast previously identified as possible sources of “reactive arthritis”; (ii) repeated triggering by such microorganisms might lead to chronic NETosis and the breakdown of tolerance to antigens released during NETosis, including citrullinated antigens targeted by RA, most specifically, autoantibodies [36].

The first hypothesis put forward to account for microbiota contribution to RA was that their antigens can be trapped in synovia [7]. It has indeed been shown that citrullinating enzyme and other major virulence factors of *Pg* are targeted to the synovium as secreted or outer-membrane-bound proteins [41,42] (Figure 1). However, it has not been yet demonstrated that these antigens are sufficient to trigger lasting arthritis. Observations made in some cases of reactive arthritis, and of Whipple’s disease, show that full triggering microorganisms, and not only their antigens, can also reach the synovia, some being still alive (albeit not replicant) [36]. Repeated translocations of live bacteria across a dysfunctional gut barrier would be much more powerful triggers of synovial long-lasting immune responses than the transient presence of bacterial antigens (which should be quickly cleared by innate cells from the synovium) is. Those translocations would fit as well or better with the frequent precession of RA by palindromic rheumatisms [36], and the frequency of flares once RA has developed. They depend on genetics and on the host response, which normally restricts the bacteremic translocation of members of the oral microbiota such as *Aa* to distant organs, thus constraining the morbidity and mortality of the host [43]. Notably, only select subtype(s) of a given species are prone to extra-oral translocation in humans [7].

The repeated translocation of nonreplicative or poorly replicative microorganisms (that might differ according to patients) from mucosal microbiota to various joints/enthesis, favored by mucosal dysbiosis associated with some HLA subtypes [32], and possibly genes of metabolism, would allow for direct interactions of those ectopic pathobionts with immune synovial cells. Those contacts, up to the internalization of some bacteria by macrophagic cells, may be sufficient to instigate autoimmunity, first focally and then systemically [6], especially following lasting epigenetic changes in long-lived synovial presenting innate cells such as embryologic-derived synovial resident macrophages, and/or stem cells induced by microbial danger signals (“trained immunity”) [39,40,44].

**Figure 2:**
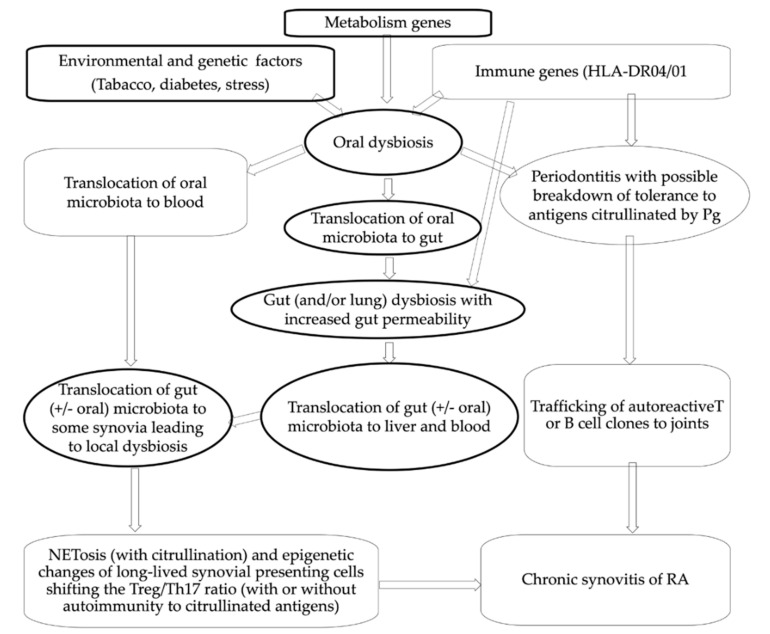
New look at oral bacterial contribution to RA.

Such periodic translocations of oral/gut bacteria to synovia might also contribute to induce local dysbiosis in some synovia/synovial fluids. Indeed, synovia are not perfectly sterile [45], and healthy synovia can harbor nonreplicative bacteria from microbiota. In normal conditions, they do not an elicit immune response because of their usual nonreplicative behavior and/or the immunosuppressive properties of synovial stromal cells when they only meet commensal bacteria (symbionts). This equilibrium can be broken when changes in the microenvironment of those cells promote their metabolic reprogramming [46], and/or when pathobionts take precedence over symbionts (dysbiosis). Translocation of excess of oral or gut pathobionts in synovia could also finally lead to NETosis (sudden release of networks of extracellular fibers, primarily composed of DNA and citrullinated peptides from neutrophils, which bind pathogens) with citrullination of self-antigens like enolase, fibrinogen, and histone, without the need for citrullination by the bacterial PAD of *Pg* [47]. Overpresentation within the synovium of citrullinated peptides by trained stromal cells or synovial-resident macrophages to B cells and T cells could then be sufficient to induce ACPAs, years before synovitis becomes detectable. Subsequent challenges by new waves of translocation of mucosal pathobionts might be required to reinforce epigenetic changes in presenting cells, and finally lead to the onset of arthritis, with or without the synthesis of RF and ACPAs (Figure 2). This scenario would account for subsequent flares of RA, and the paradoxical higher frequency of constitutional immune deficiencies in juvenile RA. This hypothesis would also fit with the otherwise puzzling association between RA and synovial metabolic backgrounds [48] (enhancing the risk of persistence of some pathobionts in synovial fluids), and the numerous clues suggesting that some bacteria from the oral cavity and/or gingiva can indirectly contribute to the pathogenesis of a subset of RA (Table 1). Notably, those oral bacteria might not be restricted to those first suspected as the most implicated in the pathogenesis of periodontitis (such as *Pg* and *Aa*).

This narrative review aims to (i) list recent evidence supporting this scenario, namely, the identification of microbiotal in the DNA in synovium of patients with RA (and of HC), including some oral bacteria, and the possibility of translocation of such bacteria from microbiota through blood, either from the oral cavity (directly or following a first step of translocation in the gut), or through an increase of gut permeability towards various gut bacteria. Such translocations might foster lasting dysbiosis in some RA synovia, and subsequent epigenetic changes in presenting cells; (ii) suggest methodologies for future works other than cross-sectional studies of periodontal microbiota in cohorts of patients with RA versus controls.

## 2. Translocation of Oral Microbiota toward Synovium through Blood May Occur Either Directly or Following Gut Colonization

### 2.1. Direct Translocation from Gingiva to Synovia

Emerging evidence suggests that periodontitis-associated pathogens can translocate to distant sites to elicit severe local and systemic pathologies [49]. Those translocations occur during transient bacteremia, which may result in bacterial colonization in remote sites [7], including synovia. This transient presence of microorganisms in blood might partly account for the increased risk of cardiovascular disorders in patients with RA [7]. Indeed, bacteria from the oral cavity first reach the circulation and can also cause low-grade inflammation in vessels, contributing to atherosclerotic cardiovascular diseases. Gut-derived microbiota on the other hand can influence host metabolism on various levels [50]. Consequently, some oral bacteria, such as *Pg*, may enter blood directly from the gingiva or dental apex (as suggested by the description of mucosal-associated invariant T cells in peri-apical lesions [51]).

### 2.2. Translocation of Some Oral Bacteria in Blood May Occur Following a First Step of Migration of Those Bacteria to Gut

Other oral bacteria, might rather reach synovia following a first step of translocation to the intestinal mucosa [52]. This may occur in conditions of oral gut barrier dysfunction [52], but also under physiological conditions. Indeed, whereas gut-to-oral microbial transmission occurs in inter- and intrapersonal manners [53], the oral–gut microbiome cross talk is bidirectional, and the oral microbiome [54] has downstream effects on the gut microbiome even in healthy persons [55].

In a seminal work in 2014, stool samples showed a significant association with samples from within the oral cavity, the strongest association being with the community types observed in saliva [56]. Although some authors previously listed *Pg* [54] as a species able to translocate from the gingiva to the large intestine, the extensive work by Schmidt et al. [56] failed to confirm this, at least when using stools as gut samples. Conversely, these authors confirmed that other species associated with periodontitis showed increased evidence for transmission from the oral cavity to the gut (*p* = 0.002), although this signal was mostly due to mildly periodontic species, including Prevotella species [56].

This may fit with the observation that periodontal disease exacerbates gut inflammation in vivo, through the expansion of oral pathobionts or pathogens (including *Klebsiella* and *Enterobacter*) that are ingested and activate Th17 in both oral and gut tissues [57,58]. Periodontitis can then supply the gut with colitogenic bacteria and pathogenic Th17, which can result in colitis, increased gut permeability, and translocation of various other bacteria through the intestinal barrier [57]. This could also contribute to RA through gut dysbiosis (which increases gut permeability), especially in patients with genetic defects causing both periodontitis and perturbations in the mechanisms underlying homeostasis in the gut [59]. Treatment-naive patients with RA displayed mildly elevated transmissibility from the oral cavity to gut across all taxa (d = 0.03; *p* = 0.01), species that were orally depleted in patients with RA showing an even markedly increased transmission to gut scores (d = 0.61; *p* = 10^−21^) [56].

In mice, intestinal barrier function is impaired before the clinical onset of arthritis [60]. In humans, serum markers associated with impaired intestinal barrier function, such as zonulin, are also increased before the onset of RA, and associated with a higher risk to develop RA later on [60]. Further works assessing blood levels of zonulin might confirm that periodontitis is associated with this impaired barrier function of gut epithelia, especially in patients with RA.

The impairment of gut wall barrier function promotes the transfer of enterobacteria and hepatotoxins to the liver through the enterohepatic circulation [61], but some bacteria from microbiota can escape the liver firewall and reach the general circulation. Excessive enterohepatic circulation can cause a transient hepatic disease [62], a phenomenon commonly observed in patients with RA, especially following treatments such as methotrexate (which modify gut homeostasis [63], but cannot always restore gut impermeability). Notably, calcitriol treatment attenuates intestinal permeability, reduces bacterial translocation, and enriches potentially beneficial gut microbiota in cirrhotic rats [64].

The imbalance in gut microbiota allowing increased microbial translocation may also concern the mycobiota in conditions sometimes overlapping with RA, such as psoriatic rheumatism and spondyloarthritis. Indeed, some fungi (pathobionts) can also cross the gut epithelia and colonize the host, whereas others (symbionts) lower the risk of other fungi and bacterial translocation [65]. For instance, compared to placebo, 12 weeks of treatment with probiotic *Saccharomyces boulardii* significantly reduced plasma levels of bacterial translocation (lipopolysaccharide-binding protein) and systemic inflammation (IL-6) in 44 HIV virologically suppressed patients [66]. 

As to RA pathogenesis, in the SKG mice model of arthritis, *Pg* oral infection affected gut microbiota dysbiosis and joint destruction via the increased generation of citrullinated peptides in the gingiva, but also in intestinal and joint tissues [67]. Importantly, fecal microbiota transplantation from *Pg*-inoculated experimental arthritis mice reproduced donor gut microbiota and resulted in severe joint destruction, with increased IL-6 and citrullinated peptide production in joint and intestinal tissues. This suggests that gut dysbiosis induced by *Pg* oral infection may be sufficient to trigger or worsen arthritis in mice [67]. This is much less sure in humans, as previous works concluded that *Pg* poorly translocates from the gingiva to stool in humans, including in patients with RA [55], so oral bacteria other than *Pg* and *Aa* might use the oral–gut–joint axis to trigger RA.

### 2.3. Translocation of Oral Bacteria in Blood Has Been Observed, including in Patients with RA

The existence of microbial populations in classically sterile locations, including the blood and synovium, is a relatively new concept, but many authors now agree about the existence of a “blood microbiome”, even in healthy people [68]. This is based on the presence of bacteria-specific DNA in the blood [68], even when robust experimental procedures were used. Although it is still not clear how such a microbiome relates to viable microbiota [69], the key phyla detected are rather consistent across studies, irrespective of the molecular method used (DNA vs. RNA) [68]. Those bacteria cannot usually be cultivated, although some may grow slowly in aerobic or anaerobic cultures even in healthy subjects [68]. In this respect, some authors advocated the use of testing a medium supplemented with a high concentration of vitamin K (1 mg/mL), and culturing at 43 °C for 24 h [70,71], to demonstrate that the bacterial DNA found in blood is not just cell-free DNA, but belongs to still alive bacteria and fungi, some being able to multiply in the erythrocytes [70,71]. 

Through the amplification and sequencing of the bacterial 16S rRNA variable region four, some authors similarly reported on the presence and identity of microbial DNA in blood samples obtained from patients with RA (both prior to and 3 months after starting treatment) [72]. Taxonomic classification revealed that the blood microbiome community in RA was distinct from that of ankylosing spondylitis, psoriatic arthritis, and the healthy state [72]. This microbiome partially normalized following treatment in some patients, especially in patients with seronegative arthritis [72]. This suggests that the systemic inflammation induced by arthritis can foster a vicious circle of further translocation of microbiota in blood.

At the phylum level, the whole blood microbiome is predominated by *Proteobacteria*, *Actinobacteria*, *Firmicutes*, and *Bacteroidetes* [68]. As many studies of blood bacterial DNA content found *Proteobacteria* DNA to be the dominant microbiome component, it was thought to originate mainly from the gut. However, some authors recently concluded that many members of the blood microbiome that were still alive were more likely to have originated from the oral or skin communities [68]. This is not surprising, as periodontal treatment, dental probing, and toothbrushing, have been shown to cause transient bacteremia. Moreover, oral bacteria from the phyla *Firmicutes* (e.g., *Streptococci*) and *Bacteroidetes* (e.g., *Porphyromonas*) can be found in cardiovascular lesions [69]. Using a method that purifies DNA from intact bacterial cells only, it has also been confirmed that in blood donated by patients with active and severe periodontal diseases, and periodontally healthy controls (HCs), 43%–52% of bacterial species in blood could be classified as oral [69], whereas in studies using total DNA, levels of *Proteobacteria* were lower, in both healthy people (17.6%) and patients with periodontitis (13.3%) [69].

### 2.4. Microbiotal DNA, including DNA from Oral Bacteria, Has Been Found in Synovia of Patients with RA and Healthy Controls

The presence of chromosomal DNA from various bacterial species was reported 20 years ago in synovial tissue from patients with diverse forms of arthritis [45,73]. As similar findings were made in HCs [72], the existence of a core synovial microbiome of nonreplicating bacteria has been postulated, whose significance is still unclear. Whether those bacteria are still alive has also not been thoroughly evaluated, although it has been shown in other tissues, such as heart valves, that alive bacteria may persist in a nonreplicative form while remaining attached to heart tissues in patients with no symptoms of infective endocarditis [74].

Those dead or alive microbiotal bacteria might have no physiological role and might be tolerated in the synovium just because of the similarities between some of their antigens and joint antigens. Their recognition by regulatory T cells specific for synovial tissues might indeed protect them from excessive immune responses in normal conditions. For instance, an amino acid motif in the the RgpA catalytic domain of *P. gingivalis* shares sequence homology with type II collagen [17].

However, as human hyaline cartilage also harbors a microbiome that differs according to the joint (knee versus hip), and the presence or not of osteoarthritis [75], it can be speculated that synovial and cartilage microbiomes could contribute to the homeostasis of joint metabolism. Indeed, in conditions such as hypoxia, human enzymes may be much less effective than enzymes of anaerobic bacteria from microbiota are. Whether genes of metabolism contribute to shape the profile of microbiota DNA found in human synovium and cartilage is a question that makes sense, especially as RA is strongly associated with the metabolic profile of synovial fluid [48], and host metabolism strongly shapes the oral microbiota [76].

As *Proteobacteria* predominate in human synovium [72], the physiological synovial microbiome might originate mainly from anaerobic bacteria from the gut. However, the presence of DNA from oral/gingival bacteria has also been reported on several occasions, either in RA synovium or in synovial fluid [77,78,79,80,81,82]. In patients with RA also diagnosed with periodontitis, identical bacterial clones (*Fusobacterium nucleatum* and *Serratia proteamaculans*) were also detected in both the synovial fluid and dental plaque samples [79]. 

### 2.5. Acquired Dysbiosis in Oral Mucosa Might Translate in Lasting Dysbiosis in Some RA Synovia, Following Translocation of Various Pathobionts

The presence of DNA of oral and gut bacteria in normal synovium and cartilage shows that the normal synovial microbiome does not induce synovitis by itself, in the same way as normal gut flora is not associated with colitis. However, transient or lasting dysbioses in the synovium or synovial fluid could contribute to RA, especially if oral pathobionts such as *Pg*, or other pathobionts from gut or lung microbiomes, deliver danger signals to long-lived presenting cells, including cells with stem cell properties, such as tissue-resident macrophages of embryologic origin [83], and/or mesenchymal stem cells of synovial tissues [44]. Epigenetic changes in those cells induced by recurrent or lasting dysbioses in the synovium might indeed be both necessary and sufficient to induce lasting arthritis. This trained immunity in some joints (but not all: hips are for instance most often preserved in RA) may be even more important than the breakdown of tolerance to citrullinated peptides (which is missing in 25% of RA cases, can be noticed in healthy people, and can antedate the onset of RA by 10 years). The overpresentation of bacterial antigens to T cells following trained immunity of synoviocytes could finally lead to a chronic imbalance between various effector T cells (Th1 and Th1) and regulatory T cells in affected synovia, without the need for recognition of a limited set of autoantigens other than citrullinated peptides. This may also explain why drugs targeting presenting cells (including B cells) and/or drugs that can correct dysbiosis in various tissues (methotrexate also impacts microbiomes [63] are much more effective at treating RA than drugs targeting T cells (e.g., ciclosporin) are. 

## 3. Methodologies Other Than Transversal Studies of Periodontal Microbiota in Cohorts of Patients with RA versus Controls Are Required

To better demonstrate that periodontal disease and/or oral bacteria contribute to the pathogenesis of some cases of RA, longitudinal studies of patients with RA would probably be much more appropriate than further cross-sectional works on the prevalence of periodontitis in RA. Those studies may ideally focus on at-risk patients (such as those sharing HLA or ACPA without any synovitis yet), even before the onset of their RA, and regardless of the presence of periodontal disease. 

### 3.1. Studies of Individuals at Risk of RA Even before the Onset of Arthritis, and Very Early RA

Such a study recently comprehensively characterized the oral microbiome in at-risk individuals (ACPA-positive, but without clinical synovitis yet). It concluded that those patients with pre-RA had dysbiotic subgingival microbiomes and increased abundance of *Pg* compared with controls [84,85]. This supports the hypothesis that the oral microbiome, and specifically *Pg*, may be important in the initiation of some cases of RA [84,85]. Notably, dysbiosis of the oral microbiome might contribute to RA independently of periodontal status, as concluded in another study, also showing that patients with early RA had enriched levels of *Prevotella pleuritidis*, *Treponema denticola*, *Porphyromonas endodontalis,* and *Filifactor alocis* and species in the *Porphyromonas* and *Fusobacterium* genera with functions linked to ornithine metabolism, glucosylceramidase, beta-lactamase resistance, biphenyl degradation, fatty acid metabolism, and 17-beta-estradiol-17-dehydrogenase metabolism [76].

### 3.2. Longitudinal Studies of Microbiota in Individual Patients with RA (before, during, and Following Flares) with Simultaneous Assessments of Synovial, Blood, Oral, and Gut Microbiota

To better guarantee that changes in oral microbiota do drive RA, longitudinal studies of those patients with early RA will be even more instructive if they encompass simultaneous studies of synovial, blood, oral, and gut microbiota. Such a methodology may confirm (or not) that the increase of RA disease activity is antedated by changes in oral and/or gut dysbiosis, followed by excessive translocations of some oral and/or gut bacteria toward synovia. A set of patients with long-lasting but unstable RA might nevertheless also be followed-up, and subgingival plaques, blood, and gut feces sampled on a regular basis (e.g., twice a month) in such patients, as translocation of oral and/or gut bacteria might still fuel RA activity long after its onset. Such a methodology recently showed that flares of RA were antedated by two weeks by a brisk increase of B cells in the blood, followed one week later by the circulation of pre-inflammatory mesenchymal cells [86] The demonstration of a wave of translocation of oral and/or gut bacteria from mucosae to synovium through blood just before B cell activation could have tremendous consequences on our understanding of RA pathogenesis. It could also show that only a minority of patients with RA may be directly driven by oral bacteria, as bacteria or other microorganisms from the gut (and perhaps other mucosae), such as *Prevotella copri* [22], possibly contribute more directly to RA than most oral bacteria do (which might be pathogenic mainly through induction of gut dysbiosis and increased gut permeability). 

### 3.3. Separate Analysis according to HLA Background and Other Parameters That May Affect Oral and Synovial Dysbiosis, Such as Smoking, Metabolic and Hormonal Status (Age and Sex), and Ongoing Treatments

As oral bacteria might contribute to only a subset of cases of RA (which is probably more a syndrome driven by various external exposures on a more homogeneous genetic background), those microbiomes should be analyzed together with cofactors that might shift oral, gut, blood, and synovial microbiota by their own, namely, gene polymorphisms, hormonal status stress, habits such as smoking or consumption of highly processed foods, and medication [87].

#### 3.3.1. Genetics

Inoculation of the oral cavity of “humanized” B6.DR1/4 mice with *Pg* results in an increase in the percentage of circulating Th17 cells, loss of bone, and an exacerbation of experimental autoimmune arthritis. The presence of the SE in the context of inoculation with *Pg* enhanced the percentage of Th17 cells, dramatically enhanced bone loss, and allowed for the generation of ACPAs not found in C57BL/6 or DBA/1 arthritic mouse serum [88]. In humans, separate analyses for patients with RA with or without SE could also tell whether oral bacteria, including *Pg,* are pathogenic and/or foster ACPAs only in patients with the HLA DR1/4 background. Other susceptibility genes should also be analyzed, including genes of metabolism. 

#### 3.3.2. Stress, Hormonal Status, and Tobacco

Stress contributes to increase gut permeability, and this may explain why it often precedes RA flares. Sex hormones also interact with the equilibrium between microbial habitats and the host immune response [89]. Therefore, longitudinal analyses of oral, gut, blood, and synovium microbiomes might be separately analyzed in males and females, and in young versus old, especially as RA is mostly a female disorder (three of four of patients are female). Analyses may also be conducted according to tobacco exposure, because smoking has a great influence on both oral and gut microbiomes and RA severity [1].

#### 3.3.3. Ongoing Treatments

Oral and gut microbiomes are highly sensitive to drugs, including antibiotics [90] and drugs used to treat RA, including methotrexate [63]. Reciprocally, some of those drugs, such as methotrexate, are metabolized by the gut microbiome, which could explain why only 50% of patients with RA are good responders to methotrexate [91]. The effects of those drugs on the composition of microbiomes can hardly be predicted at the individual level, as the follow-up of 18 patients with RA given methotrexate or another DMARD (disease-modifying antirheumatic drug) showed individual specific sharing of pre and post strains for up to 16 months [92]. However, at the group level, changes in oral, gut, blood, and synovial microbiomes should nevertheless be analyzed according to the kind of drugs used (such as methotrexate, biologics, and/or corticosteroids), and adjusted to RA activity and inflammation. Indeed, some dysbiosis within gingival or gut tissues can be reversed by treatments [2,3], and the same might hold true within blood and synovial tissues. It would not be surprising that, besides their beneficial effects on synovial inflammation (and/or dysbiosis in synovial tissue), many drugs used to treat RA can also improve gut and oral dysbiosis by reducing systemic inflammation, and lower gut permeability, leading to lower risk of further translocation of pathobionts toward the synovium. Conversely, although antibiotic treatment ameliorates invasion of the oral mucosa, it aggravates dissemination through the intestinal mucosa [93], in accordance with the observation that antibiotics usually do not improve RA. 

### 3.4. Concurrent Assessments of Immune Responses (Including Regulatory T Cell and Th17 Responses) through Transcriptomic Analyses in Gingival Sulcus, Blood, and Synovium

Both recirculating (CD69^−^), and gingiva-resident (CD69^+^) memory T cells represent an important part of the immune surveillance network in the connective tissue, maintaining periodontal homeostasis. Imbalance of subgingival bacterial communities could damage the gingival barrier, allowing bacterial antigens to gain access to the deeper connective tissue, where they activate memory T cells, leading to periodontitis [27,94]. The subset of recirculating (CD69^−^) gingival memory T cells may then also translocate to other tissues, including the synovium [27], and it might contribute to some RA flares without the need of a simultaneous new wave of translocation of oral bacteria (if some of those bacteria or their antigens remain intact in the joint for a long time following previous translocations to the synovium).

Simultaneous analyses of microbiomes and T cell responses in those various tissues might therefore show whether translocations of oral bacteria (either directly through blood, or indirectly, following a first step of translocation to the gut) are mandatory to induce each flare of RA, since homing in joints of such T cell clones specific for oral bacteria and reactivated in the oral cavity or gut may be an alternative explanation for flares. Such studies could also tell whether a defect in Tregs, or transient conversion of gingival/gut/synovial Tregs to the Th17 phenotype following excessive stimulation by oral/gut pathobionts in the synovium, contribute to RA flares, after transient translocation of oral or gut pathogens to the blood and synovium. Indeed, regulatory T cells restrict gut permeability to bacterial antigen translocation, and preserve short-chain fatty acids in experimental cirrhosis [95]. Tregs in gingival tissue and/or the dental apex might similarly prevent bacterial translocation in healthy gums, by lowering inflammation in the gingiva. As Tregs can also taper Th17 responses, this may explain why IL-17A levels were unexpectedly lower in the RA with periodontitis group but not in other patients with RA, in a transversal study where the RA plus periodontitis group also showed decreased IL-17A levels in advanced stages of periodontitis [96].

## 4. Conclusions

The moderate association of periodontal disease with RA does not imply direct causality, and only a minority of RA cases may be triggered by an immune over-response to oral bacteria. However, a growing body of evidence supports the hypothesis that RA may be a syndrome, sometimes induced by silent translocations of oral microorganisms either directly to joints, or indirectly following translocation to the gut and an increase of intestinal barrier permeability. This last possibility would fit with the observation that patients with RA have more mouth-to-gut microbial transmission than their healthy counterparts do [55].

Dysbiosis induced by repeated translocation might indeed lead to increased gut permeability and further translocation of either oral or gut bacteria from the gut to joints through blood, in patients with genetic backgrounds promoting dysbiosis in those muco-sae. Acquired dysbiosis in some synovia may finally lead to lasting epigenetic changes in long-lived synovial cells, and chronic immune responses with NETosis. This might lead to a breakdown of tolerance to citrullinated antigens shed following chronic synovial NE-Tosis, especially when pathobionts better resisting NETosis, such as *Pg*, contribute to the dysbiosis.

In other words, future prospective studies of oral, gut, blood, and synovial microbiomes in patients genetically at risk for RA, and during RA flares, could tell how often oral bacteria reach the synovia to fuel RA, either directly or following their translocation in and then through the gastrointestinal tract, where they could also increase gut permeability to other gut pathobionts.

## Data Availability

Not applicable.
